# Three cases of non‐atopic hyperkeratotic hand eczema treated with dupilumab

**DOI:** 10.1111/cod.13693

**Published:** 2020-10-01

**Authors:** Laura Loman, Gilles F.H. Diercks, Marie L.A. Schuttelaar

**Affiliations:** ^1^ Department of Dermatology University of Groningen, University Medical Center Groningen Groningen The Netherlands; ^2^ Department of Pathology University of Groningen, University Medical Center Groningen Groningen The Netherlands

**Keywords:** dupilumab, hand dermatitis, hand eczema, hyperkeratotic hand eczema, treatment

Dupilumab, a monoclonal antibody inhibiting interleukin (IL)‐4 and IL‐13 signaling, is currently approved for the treatment of atopic dermatitis (AD). Effective treatment of hand eczema with dupilumab has been reported previously in case series, two small retrospective cohorts, and one prospective observational study including atopic, irritant and vesicular hand eczema subgroups.^1^, ^2^, ^3^, ^4^ However, the effect of dupilumab on isolated hyperkeratotic hand eczema (HHE) has not yet been described.

**FIGURE 1:**
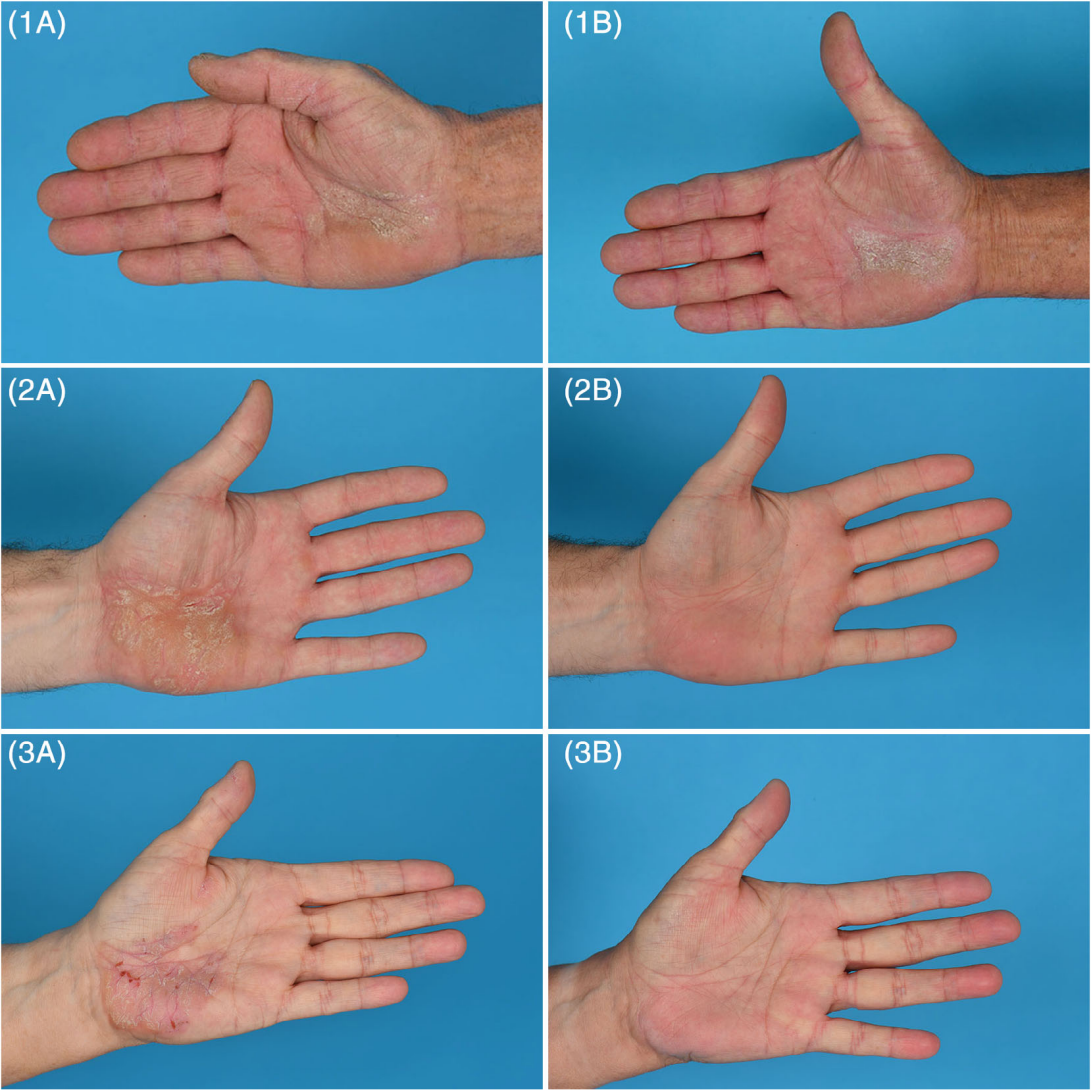
Clinical improvement of the three cases after 16 weeks of dupilumab treatment. (1A, 2A,3A) case 1, 2 and 3 at baseline, (1B, 2B, 3B) case 1, 2, 3 after 16 weeks of treatment

## METHODS

Three patients with moderate to severe HHE were treated with dupilumab 600 mg subcutaneously on day 1, followed by 300 mg subcutaneously every 14 days. A minimum washout of topical treatment of 2 weeks was applied. All patients underwent patch testing and no relevant contact allergies were detected. Concomitant fungal infections were ruled out and the diagnosis HHE was confirmed by histopathology.

Age, sex, disease duration, occupation, smoking status, treatment history, exposure to irritants, atopic comorbidities, and specific immunoglobulin E (IgE) inhalant allergens were assessed at baseline. Therapeutic response was evaluated every 4 weeks up to 16 weeks of treatment by the hand eczema severity index (HECSI),^5^ the photographic guide,^6^ clinical photographs, Quality of Life in Hand Eczema Questionnaire (QOLHEQ),^7^ and the weekly average of the number rating scale (NRS) for pain and pruritus (0–10, with 10 being the worst pruritus/pain).

## RESULTS

Two males and one female, of respectively 65, 47 and 65 years of age, were treated (Appendix S1). All of them were previously treated with ultra‐potent topical corticosteroids and at least two different systemic therapies, alitretinoin, among others. Case 1 had an inadequate response to alitretinoin, Cases 2 and 3 were intolerant to alitretinoin. None of the patients had a history of AD. Case 1 was a current smoker with 88 pack‐years and experienced occupational exposure to irritants as a bricklayer by friction and wearing gloves during part of the day. All lesional skin biopsies presented identical histopathologic features (Appendix S2).

Two patients (case 2 and 3) had already major improvement after 4 weeks and symptoms cleared completely after 16 weeks of treatment. Case 1 noticed minimal clinical improvement, however, there was an improvement in itch and quality of life (Figure 1).

## DISCUSSION

The pathogenesis of HHE remains largely unclear. A previous study on the gene and protein expression of hand eczema, including 15 patients with chronic hyperkeratotic‐fissured hand eczema showed, among others, increased proliferative cell activity indicated by Ki‐67, and a decreased expression of the terminal differentiation marker loricrin in palmar lesional skin biopsies, which was normalised following alitretinoin treatment.^8^ Another study showed an upregulation in keratinocyte host defence mechanism proteins (S100A7/S100A8/S100A9) in six patients with chronic hand eczema, including different subtypes, compared to healthy control skin.^9^ A study on protein expression in seven patients with HHE, showed also increased proliferative cell activity indicated by Ki‐67 and a strong upregulation of keratin (K)16 with, in addition, a decreased expression of loricrin in lesional palmar skin compared to perilesional skin and healthy control skin.^10^ In patients with AD treated with dupilumab a significantly reduced gene expression of K16 and MKi67, a reduced expression of S100As genes, and an increase in loricrin expression is seen after treatment.^11^ Therefore, the good effect of dupilumab on HHE could be explained by the similarities in the epidermal pathology in patients with AD and HHE, including epidermal hyperproliferation and an impaired epidermal barrier.

Lack of clinical improvement in Case 1 might be explained by his occupational activities as a bricklayer, whereby hand eczema is also caused by exposure to irritants and friction. To date, no consensus has been reached on the classification system of hand eczema. It depends on the classification system if HHE is classified as an endogenous subtype without identifiable cause^12^ or if it could have contributing identifiable causes such as exposure to irritants.^13^ However, only one case report published the successful dupilumab treatment of occupational irritant hand dermatitis^14^ However, it is possible that the role of different cytokines (including IL‐4) is affected by several other factors, such as the identity of the topical irritant or to what extent the irritant factor contribute to the etiology. Another contributing factor to the lack of clinical effect could be that the patient was smoking.

Previous literature has showed successful results of dupilumab on hand eczema in patients treated for AD^1^ and in individuals with isolated vesicular hand eczema.^2^, ^4^ In view of the good effect of dupilumab on HHE as well, it might be hypothesized that, despite the differences in phenotype, there are similarities in endotype and underlying pathogenesis with an IL‐4/IL‐13 driven inflammation between different subtypes of hand eczema. Therefore, dupilumab could be considered as an effective treatment of severe hand eczema, regardless of subtype. This should be investigated in future studies.

## CONFLICTS OF INTEREST

Dr Schuttelaar is a member of advisory boards and received consultancy fees and fees for arranging education from Sanofi‐Genzyme and Regeneron. The other authors declare no conflicts of interest.

## AUTHOR CONTRIBUTIONS

**Laura Loman:** Conceptualization; data curation; formal analysis; investigation; methodology; project administration; visualization; writing‐original draft; writing‐review and editing. **Gilles Diercks:** Visualization; writing‐review and editing. **Marie Schuttelaar:** Conceptualization; formal analysis; investigation; methodology; supervision; writing‐original draft; writing‐review and editing.

## Supporting information


**Appendix** **S1.** Patient characteristics and outcome measures.

**Appendix** **S2.** Histopathological features.
