# Supplementary material for: Three cases of non‐atopic hyperkeratotic hand eczema treated with dupilumab
Source: Contact Dermatitis. 2020 Oct 1;84(2):124–7. doi: 10.1111/cod.13693 (PMC7891406; doi:10.1111/cod.13693)
Supplement: Supplementary file 1 — Appendix S1. Patient characteristics and outcome measures. [file COD-84-124-s001.docx]

**Supplement 1
Three cases of non-atopic hyperkeratotic hand eczema treated with dupilumab**Laura Loman, Gilles F.H. Diercks, Marie L.A. Schuttelaar

**Table S1. Patient characteristics and outcome measures**

|  | **Case 1** | **Case 2** | **Case 3** |
| --- | --- | --- | --- |
| **Age^†^** | 65 | 47 | 65 |
| **Sex** | Male | Male | Female |
| **Disease duration in years** | 4 | 9 | 4 |
| **Occupation** | Bricklayer | Accountant | Retired, housewife  Previous occupation: housekeeping cleaner |
| **Smoking status^‡^** | 88 | 0 | 0 |
| **Atopic comorbidities:**  **Atopic dermatitis**  **Allergic rhinitis  Asthma** | -  -  - | -  -  - | -  +  - |
| **Specific IgE inhalant allergens** | - | - | + |
| **Positive patch test ^§^** | - | + | + |
| **Irritant contact dermatitis** | + | - | - |
| **Treatment history:**   - **Ultra-potent topical corticosteroids** - **Alitretinoin** - **Acitretin** - **Cyclosporine** - **Methotrexate** - **Azathioprine** | +  +  +  -  +  - | +  +  +  +  +  - | +  +  -  -  -  + |
| **HECSI**  **Baseline**  **Week 4  Week 16** | 14  15  9 | 26 15 1 | 25  8  0 |
| **Photographic guide**  **Baseline**  **Week 4  Week 16** | Moderate  Moderate  Moderate | Severe Moderate  Clear | Severe  Moderate  Clear |
| **QOLHEQ**  **Baseline**  **Week 4  Week 16** | 31  17  7 | 49  25  4 | 44  10  0 |
| **Average pruritus ^¶^**  **Baseline**  **Week 4  Week 16** | 5  2  0 | 7  2  0 | 8  7  0 |
| **Average pain ^¶^**  **Baseline**  **Week 4  Week 16** | 0  0  0 | 2  3  0 | 8  0  0 |

- = no, + = yes, † = in years, ‡ = in pack-years; defined as twenty cigarettes smoked per day for one year, § = positive patch test result not relevant for the diagnoses of hand eczema, ¶ = defined as the weekly average of the number rating scale (NRS) for pain and pruritus (0–10, with 10 being the worst pruritus/pain), IgE = Immunoglobulin E, HECSI = Hand Eczema Severity Index, QOLHEQ = Quality of Life in Hand Eczema Questionnaire.
