# Supplementary material for: Three cases of non‐atopic hyperkeratotic hand eczema treated with dupilumab
Source: Contact Dermatitis. 2020 Oct 1;84(2):124–7. doi: 10.1111/cod.13693 (PMC7891406; doi:10.1111/cod.13693)
Supplement: Supplementary file 2 — Appendix S2. Histopathological features. [file COD-84-124-s002.docx]

**Supplement 2
Three cases of non-atopic hyperkeratotic hand eczema treated with dupilumab**Laura Loman, Gilles F.H. Diercks, Marie L.A. Schuttelaar


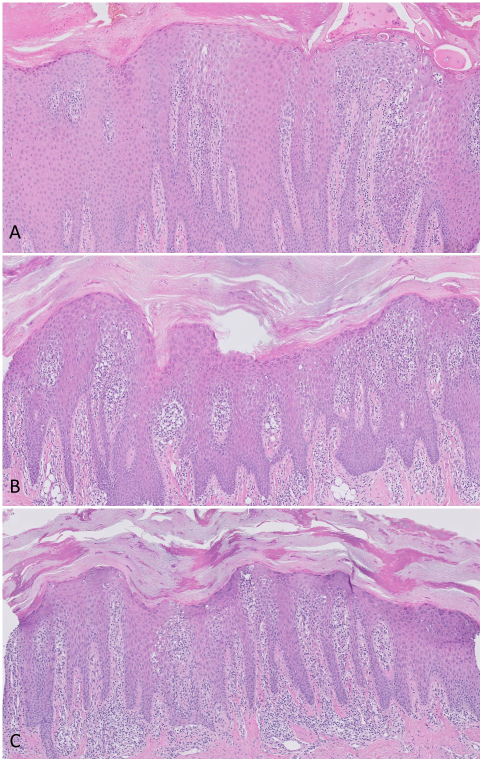


**Figure S2.** Histopathological features. (A) Case 1, (B) Case 2 and (C) Case 3. A-C, Irregular epidermal hyperplasia, a preserved granular layer, hyperkeratosis and parakeratosis, spongiosis with exocytosis of lymphocytes, and a superficial lymfohistiocytic infiltrate. Hematoxylin-eosin stain; original magnification: x100.
